# The impact of kidney function in patients on antithrombotic therapy: a post hoc subgroup analysis focusing on recurrent bleeding events from the AFIRE trial

**DOI:** 10.1186/s12916-022-02268-6

**Published:** 2022-02-25

**Authors:** Kunihiko Matsui, Satoshi Yasuda, Koichi Kaikita, Masaharu Akao, Junya Ako, Tetsuya Matoba, Masato Nakamura, Katsumi Miyauchi, Nobuhisa Hagiwara, Kazuo Kimura, Atsushi Hirayama, Hisao Ogawa

**Affiliations:** 1 Department of General Medicine and Primary Care, Kumamoto University Hospital, 1-1-1 Honjo, Chuo-ku, Kumamoto, Kumamoto 860-8556 Japan; 2 Department of Cardiovascular Medicine, Tohoku University Graduate School of Medicine, 1-1 Seiryo-machi, Aoba-ku, Sendai, Miyagi 980-8574 Japan; 3 National Cerebral and Cardiovascular Center, 6-1 Kishibe Shimmachi, Suita, Osaka 564-8565 Japan; 4 Division of Cardiovascular Medicine and Nephrology, Department of Internal Medicine, Faculty of Medicine, University of Miyazaki, 5200 Kihara, Kiyotake, Miyazaki 889-1692 Japan; 5 Department of Cardiovascular Medicine, Graduate School of Medical Sciences, Kumamoto University, 1-1-1 Honjo, Chuo-ku, Kumamoto, Kumamoto 860-8556 Japan; 6 Department of Cardiology, National Hospital Organization Kyoto Medical Center, 1-1 Mukaihata-cho, Fukakusa, Fushimi-ku, Kyoto, Kyoto 612-8555 Japan; 7 Department of Cardiovascular Medicine, Kitasato University School of Medicine, 1-15-1 Kitasato, Minami-ku, Sagamihara, Kanagawa 252-0373 Japan; 8 Department of Cardiovascular Medicine, Faculty of Medical Sciences, Kyushu University, 3-1-1 Maidashi, Fukuoka, Fukuoka 812-8582 Japan; 9 Division of Cardiovascular Medicine, Toho University Ohashi Medical Center, 2-22-36, Ohashi, Meguro-ku, Tokyo, 153-8515 Japan; 10 Department of Cardiovascular Medicine, Juntendo Tokyo Koto Geriatric Medical Center, 3-3-20 Shinsuna, Koto-ku, Tokyo, 136-0075 Japan; 11 Department of Cardiology, Tokyo Women’s Medical University, 8-1, Kawada-cho, Shinjuku-ku, Tokyo, 162-8666 Japan; 12 Cardiovascular Center, Yokohama City University Medical Center, 4-57, Urafune-cho, Minami-ku, Yokohama, Kanagawa 232-0024 Japan; 13 Department of Cardiology Osaka Police Hospital, 10-31 Kitayama-cho, Tennouji-ku, Osaka City, Osaka 543-0035 Japan; 14 Kumamoto University, 2-39-1 Kurokami, Chuo-ku, Kumamoto, 860-8555 Japan

**Keywords:** Kidney function, Estimated glomerular filtration rate, Creatinine clearance, Antithrombotic therapy, Non-vitamin K antagonistic oral anticoagulants, Recurrent event, Bleeding event, Thrombotic event, Atrial fibrillation

## Abstract

**Background:**

The success of antithrombotic therapies is assessed based on thrombotic and bleeding events. Simultaneously assessing both kinds of events might be challenging, and recurrent bleeding events are often ignored. We tried to confirm the effects of kidney function on outcome events in patients undergoing antithrombotic therapy.

**Methods:**

As a post hoc subgroup analysis of the Atrial Fibrillation and Ischemic Events with Rivaroxaban in Patients with Stable Coronary Artery Disease (AFIRE) trial, a randomized clinical trial with a median follow-up of 36 months, patients were divided into high and low estimated glomerular filtration rate (eGFR) groups with a cutoff value of 50 mL/min. The cumulative incidence of bleeding and crude incidence of recurrent bleeding per 100 patient-years were calculated. We used the Cox regression model with multiple failure time data for recurrent bleeding events.

**Results:**

Among 2092 patients, 1386 (66.3%) showed high eGFR. The cumulative bleeding events per 100 patients at 1 year were 5.4 and 6.2 in the high and low eGFR groups, respectively. The difference continued to increase over time. The hazard ratio for time to the first bleeding event in the high eGFR group was 0.875 (95% confidence interval 0.701–1.090, *p* = .234) and that for the first composite event was 0.723 (95% confidence interval 0.603–0.867, *p* < .000). The recurrent bleeding events per 100 person-years were 11.3 and 15.3 in the high and low eGFR groups, respectively, with a rate ratio of 0.738 (95% confidence interval 0.615–0.886, *p* = .001). During the observation period, the risk of bleeding changed with time. It peaked soon after the study enrollment in both groups. It decreased continuously in the high eGFR group but remained high in the low eGFR group.

**Conclusions:**

We reaffirmed that kidney function affects bleeding events in patients on antithrombotic therapy, considering recurrent events. Patients should have detailed discussions with physicians regarding the possible bleeding events when continuing antithrombotic therapy, especially in patients with decreased kidney function.

**Trial registration:**

UMIN Clinical Trials Registry, UMIN000016612. ClinicalTrials.gov, NCT02642419. Registered on 21 October 2015.

**Supplementary Information:**

The online version contains supplementary material available at 10.1186/s12916-022-02268-6.

## Background

A vast majority of antithrombotic therapies are available in the market. They have been usually assessed based on thrombotic events for efficacy and based on bleeding events for safety. These events are opposite to each other due to the nature of antithrombotic drugs. Additionally, the frequency and effect on prognosis are distinct. Fatal bleeding cases are relatively rare, while cases of nuisance bleedings are common, and their effect on the prognosis is generally limited [1]. In contrast, ischemic events are relatively less frequent but have a significant effect on the prognosis in the form of irreversible harm and often permanent disability.

Bleeding is common and can influence adherence to therapy or result in its disruption [2, 3]. Furthermore, even its severity might vary for each case, and the discontinuation of these drugs is known to increase the risk of thrombotic events [4]. Some patients who resume antithrombotic therapy may also develop bleeding events again. A consensus report recommended an individualized early resumption of antithrombotic therapy after bleeding events [4].

In addition to expert opinion, those recommendations were primarily based on previous studies. However, their data should be interpreted with caution due to concerns regarding the study settings. For example, composite endpoints are frequently used in clinical studies. However, they have been frequently criticized because each outcome event has a different effect on the overall health [5]. Furthermore, especially for antithrombotic therapy, most previous studies included only the first event as the endpoint [6], and the recurrent bleeding events were often ignored.

When considering therapy for a patient, the individual patient’s characteristics are also important. Although physicians attempt to assess each patient’s risk, the heterogeneity of patient characteristics makes applying a unified quantitative assessment of the absolute benefits and risks challenging [7]. For example, the prevalence of atrial fibrillation (AF) increases with decreasing kidney function [8], and patients with AF and chronic kidney disease have a high risk of both thromboembolic and hemorrhagic complications [9]. Meanwhile, non-vitamin K antagonistic oral anticoagulants (NOACs) are excreted mainly by the kidneys, and therefore, kidney function affects the outcome events in patients on NOACs [10].

In this study, we investigated the effects of kidney function on bleeding and thrombotic cardiovascular events, including recurrent bleeding in patients on antithrombotic therapy with rivaroxaban who were enrolled in the Atrial Fibrillation and Ischemic Events with Rivaroxaban in Patients with Stable Coronary Artery Disease (AFIRE) trial [11].

## Methods

### Study design and patients

This study was a post hoc subgroup analysis of the data acquired from the AFIRE trial; the study design and primary outcomes of the AFIRE trial have been published previously [11]. Briefly, the AFIRE trial enrolled 2215 patients from 294 centers with stable coronary artery disease with AF, and patients were randomly prescribed rivaroxaban monotherapy or rivaroxaban and antiplatelet agent combination therapy. The primary finding was that rivaroxaban monotherapy was non-inferior to combination therapy for a composite of stroke, systemic embolism, myocardial infarction, unstable angina requiring revascularization, or death. Furthermore, monotherapy was superior to combination therapy in terms of major bleeding.

In addition to the primary analysis stated above, other subgroup analyses have also been described previously [12]. The study protocol of the AFIRE trial itself was approved by the institutional review board (IRB) at each participating institution, and the study was performed in line with the Declaration of Helsinki. Of the 294 study centers, 61 did not have their own IRB, and they used the approval from the central ethics review board. All enrolled patients provided written informed consent.

### Outcomes

In this report, we focused on any bleeding events and ischemic cardiovascular events, including myocardial infarction, unstable angina requiring revascularization, ischemic stroke, systemic embolism, and death due to any cause. We also included recurrent bleeding events observed in each patient during the observation period. However, in terms of ischemic cardiovascular events, we only included the first event in each patient. We evaluated these outcomes according to the kidney function as estimated glomerular filtration rate (eGFR), represented by creatinine clearance (CrCl) as estimated using the Cockcroft–Gault (CG) equation. We focused on patients whose eGFR data were available at enrollment in the AFIRE trial (*n* = 2092). The overall median follow-up was 36 months.

### Statistical analysis

All baseline characteristics were assessed at the time of study enrollment. We divided the patients into high and low eGFR groups (eGFR ≥ 50 and < 50 mL/min, respectively) at the time of study enrollment, the cutoff point was determined according to the criteria indicated in the Japanese drug package insert. Continuous variables were compared using unpaired *t*-test, and the categorical variables were compared using the chi-square test or Fisher’s exact test, as appropriate.

Cumulative incidence of bleeding over time was calculated for the two groups. In this analysis, we considered the first bleeding event and death from any cause as competing risks [13–15]. Additionally, we performed a similar analysis with ischemic cardiovascular events, and the first event between bleeding and the ischemic cardiovascular event was considered for analysis.

We performed time-to-first event analysis for bleeding events as well as the first event analysis for composite endpoints of bleeding events, ischemic cardiovascular events, and death. We also adjusted for the differences in the patients’ baseline characteristics.

The crude incidence of recurrent bleeding per 100 patient-years of the follow-up period was calculated by dividing the total number of bleeding events in each eGFR group. The confidence intervals (CIs) were calculated using the quadratic approximation to the Poisson log-likelihood for the log-rate parameter [16]. From these results, the consequent incidence ratios were obtained [17]. Additionally, we used the negative binomial distribution model to modify an estimated rate ratio by assessing the wide variations in patients with respect to their risks of recurrent bleeding. This model used the backward selection method to select the independent covariates, including personal characteristics. Furthermore, we extended the composite of recurrent events to include all bleeding and ischemic cardiovascular events as well as death, i.e., we counted death as an additional event. The consequent rate ratio was the effect of eGFR on the composite of these events.

Since bleeding events might have occurred two or more times in a patient during the study period, we used the Cox regression model with multiple failure time data for bleeding events. We considered not only the first bleeding event but also the subsequent events. In this analysis, we assume that the hazard ratio may change with time; however, time was measured from study enrollment and was independent of the time the last event occurred. For the measured time, we used the time to each event from the time of the previous event using the conditional risk set model [18, 19] and estimated the course with the backward selection method to select independent covariates from the same variables in the previous negative binomial distribution model.

### Sensitivity analysis

In this study, we have employed eGFR, as estimated by the CG equation, and used the cutoff point of 50 mL/min. However, other methods such as the Chronic Kidney Disease Epidemiology Collaboration (CKD-EPI) equation are commonly used to estimate GFR, and their accuracy is validated well in Western countries. Furthermore, the CG equation is said to overestimate GFR and exhibit large variability compared with the CKD-EPI equation [20]. As a sensitivity analysis, we also employed the CKD-EPI equation to estimate GFR, and two groups were distinguished by the cutoff at 50 mL/min/1.73 m^2^ to compare the estimated risk of bleeding events during the observation period. Moreover, we have also employed an additional cutoff point at 45 mL/min/1.73 m^2^ and 60 mL/min/1.73 m^2^ as estimated by the CKD-EPI equation since these points are used to define the CKD stage, and they are the closest lower and higher points to 50 mL/min/1.73 m^2^. We compared these results with the cutoff points at 45 mL/min and 60 mL/min estimated by the CG equation. From these sensitivity analyses, we have attempted to determine the robustness of our results, by different GFR estimation methods and through different cutoff points.

All tests were two-sided, and *p* values < .05 were considered to indicate a statistically significant difference. All analyses were performed using Stata SE v15.1 (Stata Corp LLC, College Station, TX, USA).

## Results

### Patient background data

Among the 2092 patients, the high eGFR group included 1386 patients (66.3%; mean eGFR 74.3 ± 21.8 mL/min), whereas the low eGFR group included 706 patients (33.7%; mean eGFR 38.7 ± 7.9 mL/min) (Table 1). There were several differences between the two groups. For example, the high eGFR group had younger patients (71.4 ± 7.6 vs. 80.3 ± 5.8 years, respectively, *p* < .001), more male patients (85.6% vs. 66.0%, respectively, *p* < .001), and higher hemoglobin level (13.9 ± 1.6 vs. 12.6 ± 1.7 g%, respectively, *p* < .001) than the low eGFR group. In contrast, the low eGFR group showed higher rates of heart failure (30.3% vs. 47.6%, respectively, *p* < .001) and kidney dysfunction (0.3% vs. 1.8%, respectively, *p* < .001) than the high eGFR group. Additionally, the CHADS2 score was higher in the low eGFR group than in the high eGFR group (median, 2.0 vs. 3.0, respectively, *p* < .001). There were no differences in terms of the details of the prescribed drugs at study enrollment between the two groups, except for the initial dose of rivaroxaban based on the study protocol.

**Table 1:** Clinical characteristics of the patients at study enrollment

	Estimated glomerular filtration rate (mL/min)		Total, *N* = 2092	*P* value
	≥ 50, *N* = 1386 (66.3%)	< 50, *N* = 706 (33.7%)		
Age, years				
Mean (SD)	71.4 (7.6)	80.3 (5.8)	74.4 (8.2)	< .001
Median (Q1, Q3)	72.0 (67.0, 77.0)	81.0 (77.0, 84.0)	75.0 (69.0, 80.0)	
Male	1186 (85.6%)	466 (66.0%)	1652 (79.0%)	< .001
BMI, kg/m^2^				
Mean (SD)	25.4 (3.6)	22.7 (3.2)	24.5 (3.7)	< .001
Median (Q1, Q3)	24.9 (23.1, 27.3)	22.5 (20.5, 24.7)	24.2 (22.0, 26.6)	
BMI > 24.5 kg/m^2^	791 (57.1%)	207 (29.3%)	998 (47.7%)	< .001
SBP				
Mean (SD)	126.9 (15.4)	124.8 (16.9)	126.2 (15.9)	.004
Median (Q1, Q3)	127.0 (117.0, 136.0)	125.0 (113.0, 135.0)	126.0 (116.0, 136.0)	
SBP > 140 mmHg	308 (22.2%)	143 (20.3%)	451 (21.6%)	.301
DBP				
Mean (SD)	72.8 (11.5)	68.5 (11.8)	71.4 (11.8)	< .001
Median (Q1, Q3)	72.0 (65.0, 80.0)	69.0 (60.0, 77.0)	70.0 (63.0, 80.0)	
Estimated glomerular filtration rate				
Mean (SD)	74.3 (21.8)	38.7 (7.9)	62.3 (24.9)	< .001
Median (Q1, Q3)	68.6 (59.1, 82.8)	39.7 (33.7, 45.2)	58.9 (45.0, 74.6)	
Hemoglobin				
Mean (SD)	13.9 (1.6)	12.6 (1.7)	13.5 (1.7)	< .001
Median (Q1, Q3)	14.0 (13.0, 15.0)	12.6 (11.4, 13.8)	13.6 (12.4, 14.7)	
Current smoker	207 (14.9%)	70 (9.9%)	277 (13.2%)	.001
Type of AF				
Permanent	456 (32.9%)	217 (30.7%)	673 (32.2%)	.206
Paroxysmal	728 (52.5%)	366 (51.8%)	1094 (52.3%)	
Persistent	202 (14.6%)	123 (17.4%)	325 (15.5%)	
Comorbid conditions				
Hypertension	1201 (86.7%)	593 (84.0%)	1794 (85.8%)	.100
Diabetes	625 (45.1%)	253 (35.8%)	878 (42.0%)	< .001
Dyslipidemia	985 (71.1%)	465 (65.9%)	1450 (69.3%)	.015
Angina	857 (61.8%)	457 (64.7%)	1314 (62.8%)	.195
Heart failure	420 (30.3%)	336 (47.6%)	756 (36.1%)	< .001
Liver dysfunction	29 (2.1%)	8 (1.1%)	37 (1.8%)	.116
Kidney dysfunction	4 (0.3%)	13 (1.8%)	17 (0.8%)	< .001
Bleeding dysfunction	16 (1.2%)	14 (2.0%)	30 (1.4%)	.132
CHADS2 score				
0	5 (0.4%)	0 (0.0%)	5 (0.2%)	< .001
1	376 (27.1%)	58 (8.2%)	434 (20.7%)	
2	497 (35.9%)	240 (34.0%)	737 (35.2%)	
3	311 (22.4%)	218 (30.9%)	529 (25.3%)	
4	135 (9.7%)	124 (17.6%)	259 (12.4%)	
5	52 (3.8%)	51 (7.2%)	103 (4.9%)	
6	10 (0.7%)	15 (2.1%)	25 (1.2%)	
Mean (SD)	2.3 (1.1)	2.9 (1.2)	2.5 (1.2)	< .001
Median (Q1, Q3)	2.0 (1.0, 3.0)	3.0 (2.0, 4.0)	2.0 (2.0, 3.0)	
Past history				
Stroke	178 (12.8%)	122 (17.3%)	300 (14.3%)	.006
Transient ischemic attack	34 (2.5%)	11 (1.6%)	45 (2.2%)	.182
Myocardial infarction	468 (33.8%)	262 (37.1%)	730 (34.9%)	.129
Aortic aneurism	45 (3.2%)	26 (3.7%)	71 (3.4%)	.603
Systemic thrombosis	7 (0.5%)	3 (0.4%)	10 (0.5%)	.802
Deep vein thrombosis	9 (0.6%)	4 (0.6%)	13 (0.6%)	.820
Pulmonary embolism	5 (0.4%)	2 (0.3%)	7 (0.3%)	.772
Peripheral artery disease	64 (4.6%)	68 (9.6%)	132 (6.3%)	< .001
Other ischemic diseases	127 (9.2%)	57 (8.1%)	184 (8.8%)	.405
Other bleeding diseases	33 (2.4%)	22 (3.1%)	55 (2.6%)	.320
Intervention				
PCI/CABG	1047 (75.5%)	555 (78.6%)	1602 (76.6%)	.117
Others	186 (13.4%)	74 (10.5%)	260 (12.4%)	.054
Drugs				
Monotherapy	700 (50.5%)	353 (50.0%)	1053 (50.3%)	.827
Combination therapy	686 (49.5%)	353 (50.0%)	1039 (49.7%)	
Antiplatelet drug				
Aspirin	506 (36.5%)	253 (35.8%)	759 (36.3%)	.762
Clopidogrel	171 (12.3%)	99 (14.0%)	270 (12.9%)	.277
Prasugrel	14 (1.0%)	4 (0.6%)	18 (0.9%)	.299
Ticlopidine	2 (0.1%)	2 (0.3%)	4 (0.2%)	.491
Anticoagulant drug				
Rivaroxaban	1379 (99.5%)	697 (98.7%)	2076 (99.2%)	.056
10 mg^a^	357 (25.9%)	598 (84.7%)	955 (46.0%)	< .001
15 mg^a^	1022 (73.7%)	97 (13.7%)	1119 (53.5%)	
Warfarin	3 (0.2%)	0 (0.0%)	3 (0.1%)	.216
Dabigatran	1 (0.1%)	0 (0.0%)	1 (0.0%)	.475
Apixaban	3 (0.2%)	3 (0.4%)	6 (0.3%)	.399
Edoxaban	0 (0.0%)	2 (0.3%)	2 (0.1%)	.047
Total number of antiplatelet and/or anticoagulant drugs				
0	0 (0.0%)	3 (0.4%)	3 (0.1%)	.073
1	682 (49.2%)	339 (48.0%)	1021 (48.8%)	
2	511 (36.9%)	254 (36.0%)	765 (36.6%)	
3	189 (13.6%)	105 (14.9%)	294 (14.1%)	
4	4 (0.3%)	5 (0.7%)	9 (0.4%)	
Proton pump inhibitor	848 (61.2%)	435 (61.6%)	1283 (61.3%)	.848

*AF* Atrial fibrillation, *BMI* Body mass index, *CABG* Coronary artery bypass grafting, *DBP* Diastolic bleed pressure, *PCI* Percutaneous coronary intervention, *SBP* Systolic bleed pressure, *SD* Standard deviation

^a^From 2074 patients with available data regarding the initial dosage of rivaroxaban

### Incidence of outcome events

During the study period, up to 10 episodes of bleeding were observed per patient, and there were no differences in the bleeding incidence between the two groups (*p* = .546) (Table 2). There were 232 (16.7%) patients with at least one bleeding episode in the high eGFR group compared with 131 (18.6%) patients in the low eGFR group (*p* = .300). We observed up to 3 severe bleeding episodes, as defined by the International Society on Thrombosis and Haemostasis criteria, and the incidence of severe bleeding between the two groups (*p* = .096) did not differ significantly. The incidence of the other outcome events between the two groups did not differ significantly, except for systemic embolism and death from any cause, which was higher in the low eGFR group.

**Table 2:** Outcome events according to estimated glomerular filtration rate

	Estimated glomerular filtration rate (mL/min)		Total, *N* = 2092	*P* value
	≥ 50, *N* = 1386	< 50, *N* = 706		
Patients no. of bleeding				
0	1154 (83.3%)	575 (81.4%)	1729 (82.6%)	.546
1	171 (12.3%)	90 (12.7%)	261 (12.5%)	
2	42 (3.0%)	26 (3.7%)	68 (3.3%)	
3	14 (1.0%)	9 (1.3%)	23 (1.1%)	
4	2 (0.1%)	3 (0.4%)	5 (0.2%)	
5	1 (0.1%)	1 (0.1%)	2 (0.1%)	
6	0 (0.0%)	1 (0.1%)	1 (0.0%)	
7	1 (0.1%)	0 (0.0%)	1 (0.0%)	
9	1 (0.1%)	0 (0.0%)	1 (0.0%)	
10	0 (0.0%)	1 (0.1%)	1 (0.0%)	
Severe bleeding				
0	1336 (96.4%)	669 (94.8%)	2005 (95.8%)	.096
1	45 (3.2%)	30 (4.2%)	75 (3.6%)	
2	4 (0.3%)	7 (1.0%)	11 (0.5%)	
3	1 (0.1%)	0 (0.0%)	1 (0.0%)	
Hemorrhagic stroke	11 (0.8%)	6 (0.8%)	17 (0.8%)	.892
Any bleeding	232 (16.7%)	131 (18.6%)	363 (17.4%)	.300
Ischemic stroke	26 (1.9%)	21 (3.0%)	47 (2.2%)	.109
Myocardial infarction	11 (0.8%)	9 (1.3%)	20 (1.0%)	.285
Unstable angina requiring revascularization	17 (1.2%)	12 (1.7%)	29 (1.4%)	.381
Systemic embolization	0 (0.0%)	3 (0.4%)	3 (0.1%)	.015
All-cause death	42 (3.0%)	69 (9.8%)	111 (5.3%)	< .001

### Cumulative incidence of bleeding

**Fig. 1:**
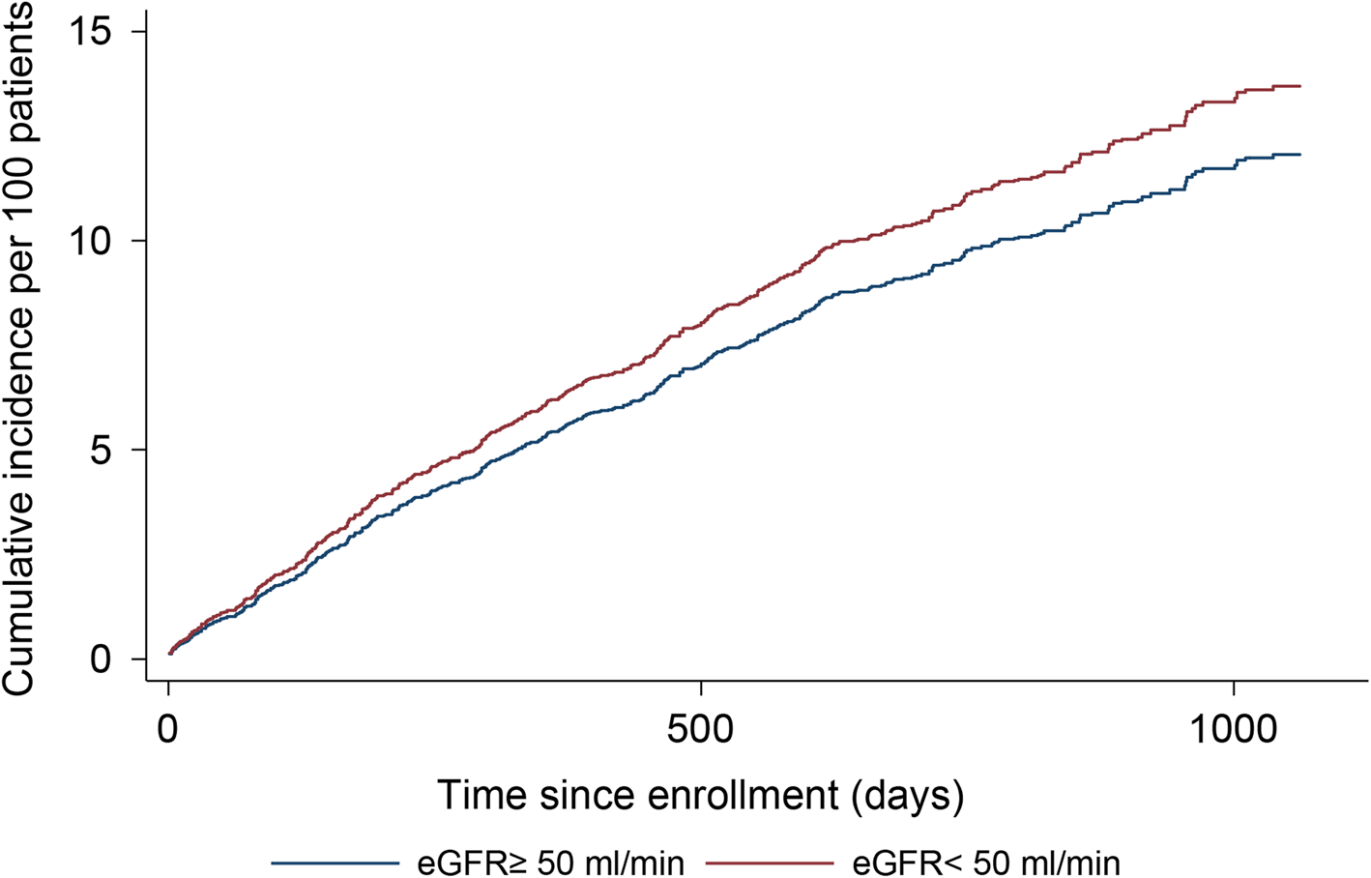
Estimated cumulative number of bleeding events per 100 patients. Time since enrollment (days)

Figure 1 illustrates the cumulative number of bleeding events per 100 patients in the two groups over time, while death was considered a competing risk. By the end of the first year, the cumulative episodes of bleeding per 100 patients were 5.4 in the high eGFR group and 6.2 in the low eGFR group (difference of 0.8 per 100 patients). Beyond 1 year, the difference increased persistently (1.3 at 2 years and 1.7 at 3 years). Similarly, the cumulative number of bleeding and ischemic cardiovascular events per 100 patients was also calculated. By the end of the first year, the cumulative events per 100 patients were 6.4 in the high eGFR group and 7.8 in the low eGFR group (a difference of 1.4 per 100 patients). Beyond 1 year, the difference increased persistently (2.3 at 2 years and 2.9 at 3 years).

### Estimation of the effect of the difference in eGFR

Table 3 summarizes the estimated effect of eGFR difference on all event outcomes. The hazard ratio in the high eGFR group compared to that in the low eGFR group was 0.875 [95% confidence interval (CI) 0.701–1.090, *p* = .234] for the first bleeding event and 0.723 (95% CI 0.603–0.867, *p* = .000) for the first composite events after adjustments. In the high and low eGFR groups, there were 314 and 198 bleeding events over 2783.4 and 1294.7 person-years of follow-up, respectively. Therefore, the bleeding rate per 100 person-years was 11.3 in the high eGFR group and 15.3 in the low eGFR group, with a rate ratio of 0.738 (95% CI 0.615–0.886, *p* = .0009) for recurrent bleeding as determined by the Poisson regression. The negative binomial regression model yielded a rate ratio of 0.860 (95% CI 0.680–1.088, *p* = .208) for the high eGFR group than that for the low eGFR group. When extending the composite outcomes other than bleeding events, the rate ratios remained constantly lower than those of the previous models in the high eGFR and low eGFR groups. The recurrent bleeding event analysis revealed larger differences than the time-to-first event analysis in the same composite outcome settings. Also, we assured that the effect of the interaction term between kidney function and the number of antithrombotic agents for all outcome analyses above was not significant.

**Table 3:** Comparison of the effect of estimated glomerular filtration rate difference among different endpoints

	Effect due to estimated glomerular filtration rate, 50 mL/min	95% CI	*P* value
Hazard ratio for the first bleeding			
Unadjusted	0.830	0.670–1.028	.088
Adjusted	0.875	0.701–1.090	.234
Hazard ratio for the first event among the composite of bleeding, ischemic cardiovascular event, and any cause of death			
Unadjusted	0.677	0.567–0.809	.000
Adjusted	0.723	0.603–0.867	.000
Rate ratios for recurrent bleeding			
Poisson (unadjusted)	0.738	0.615–0.886	.001
Negative binomial (adjusted)	0.860	0.680–1.088	.208
Rate ratios for composite of recurrent bleeding, the first ischemic cardiovascular event, and any cause of death			
Poisson (unadjusted)	0.616	0.531–0.716	.000
Negative binomial (adjusted)	0.742	0.615–0.896	.002

*CI* Confidential interval

**Fig. 2:**
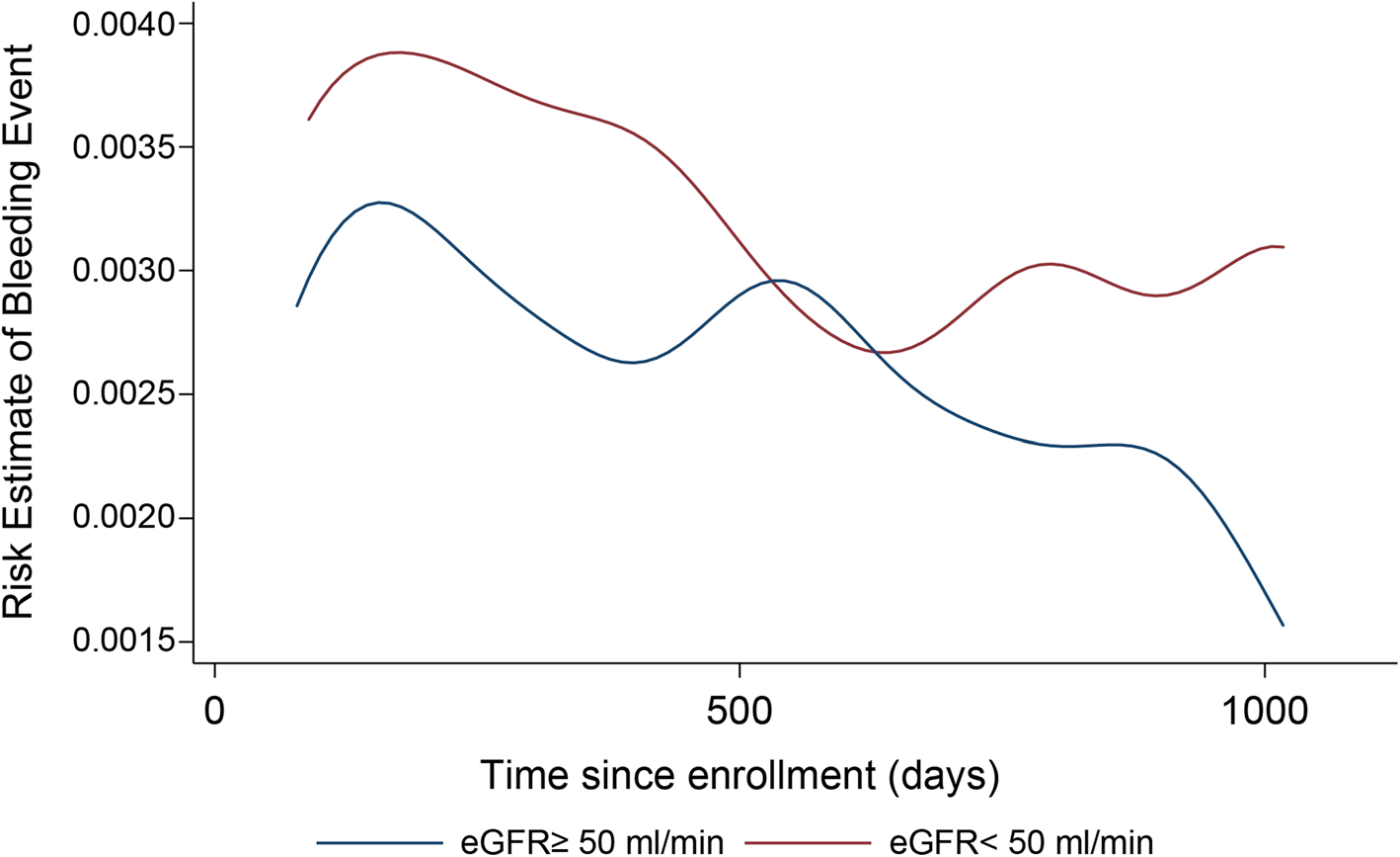
Estimated risk of bleeding events, Cockcroft–Gault equation at 50 mL/min. Time since enrollment (days)

The curves for the risk estimate of bleeding events over time stratified according to eGFR reflect the events’ dynamics (Fig. 2). Visual inspection showed a change in the risk of recurrent bleeding over the study period. It peaked soon after the study enrollment in both groups and decreased continuously in the high eGFR group; however, it remained high in the low eGFR group. The interaction term between antithrombotic therapy (monotherapy vs. combination therapy) and kidney function showed a significant effect in the models for the recurrent events (*p* = 0.042 for the model of recurrent bleeding events), but none for the first event model. In the recurrent event models, the therapy difference had a large impact on the high eGFR group.

### Sensitivity analysis

The time course of the estimated risk of bleeding events for the two groups divided at 50 mL/min/1.73 m^2^, as estimated by the CKD-EPI equation, showed similar results with that from the CG equation at the cutoff point of 50 mL/min (Fig. 2, Fig. S1).

At the cutoff point of 45 mL/min/1.73 m^2^, as estimated by the CKD-EPI equation, both groups showed a peak soon after the observation was commenced, and another higher peak was observed in the low eGFR group, afterward. On the other hand, the risk of the high eGFR group decreased continuously. The low eGFR group had a higher bleeding risk than the high-eGFR group throughout the observation period. The shapes of these curves were similar to those estimated by the CG equation at the cutoff point of 45 mL/min (Figs. S2 and S3). At the cutoff point of 60 mL/min/1.73 m^2^, as estimated by the CKD-EPI equation, the estimated bleeding risk had decreased gradually, and increased afterwards in the low eGFR group, while it had decreased from the peak in the high eGFR group. Although the high eGFR group showed a peak of bleeding risk following enrollment, the shapes of the curves were similar between the different estimation methods for GFR (Figs. S4 and S5). Between the different estimation methods for GFR at the same cutoff point, a similar time course of the bleeding risk was also observed.

## Discussion

In this study, we demonstrated the effects of kidney function, represented by eGFR, on the outcome events in patients from the AFIRE trial with stable coronary artery disease who were on antithrombotic therapy. We found that eGFR < 50 mL/min was related to a high outcome incidence, and the bleeding risk remained high over time in these patients.

Our findings are consistent with and extended the results of previous studies. Adverse medication-related outcomes in the studies for chronic kidney disease patients could be classified as those leading to kidney damage directly and other metabolic complications such as hyperkalemia and bleeding events [21]. In patients suffering from chronic kidney disease and receiving oral anticoagulant therapy, the reduction in thrombotic risk outweighs the bleeding risk in most cases and should be considered based on the balance between the benefits and risks [22]. For each type of thrombotic and bleeding event, composite endpoints are frequently used to demonstrate the summarized effect of the intervention. Although there are advantages to using composite outcomes, such as statistical power, we should also highlight their rationale. Composite outcomes are based on the assumption that a similar direction of the effect of the intervention will occur for each component of an aggregate outcome [5]. However, composite outcomes may include heterogeneous outcome endpoints with different magnitudes or directions as well as clinical impacts. In some studies, the newly proposed composite variables, such as net clinical benefits and net adverse clinical events, have also been used [23, 24]. Those variables can include both bleeding and thrombotic events, and they can be easily quantified as the summarized measures. Combining endpoints with different types of endpoints and directions can be problematic because the clinical effect of each event varies along with its severity and frequency. Therefore, we should examine the details of a composite event in a clinical context. Weighting methods have also been suggested to overcome these challenges [25, 26]. Each type of outcome event is assigned a specific weight based on the clinical effect on a patient. However, the weight for a type of outcome might not be consistent between patients and studies [27]. These findings suggest that although these new methods are promising, they are still under development for clinical use [28].

The results of the present study can be interpreted as not only an extension of the original AFIRE trial but also as complementary to that study. Our present results further explain the effect of kidney function. We also performed the analysis for recurrent bleeding events. Including recurrent events has been reported to result in greater power and more accurate estimation of the risks [29]. We added the first thrombotic cardiovascular events to the bleeding events to confirm the change in direction and magnitude. Although they should be interpreted with caution for the abovementioned composite outcomes, the comparable and consistent results demonstrated the effects of kidney function on the outcome events. Without including bleeding events, these results would have underestimated the effects of kidney function on the outcome events. The effect on the outcomes was quite large, as evident from the difference of incidence between the groups. It is expected that a bleeding event could have influenced the decision to continue the drugs and that discontinuation of drugs would consequently be related to the thrombotic events. However, our findings for the effect of kidney function on the outcomes would be consistent.

In our study, the bleeding risk increased in both groups after enrollment. However, the risk in the low eGFR group was higher than that in the high eGFR group. Over time, the bleeding risk in both groups decreased, but that in the low eGFR group remained high during this study period. It has been reported that the risk of bleeding in patients on anticoagulant therapy peaks immediately after the initiation of therapy [30]. However, the results from the said study might be biased due to the enrollment of prevalent drug users, in addition to the disease severity [31]. In our study, both groups had similar drug usage in addition to the other background characteristics at enrollment, which would have had little effect on our results. Therefore, we should consider resuming oral anticoagulants soon after bleeding events, especially in patients with preserved eGFR. In contrast, detailed discussions and considerations between patients and physicians are necessary, especially in patients with decreased eGFR. These findings are consistent with and extend expert consensus report, which recommended resuming oral antithrombotic therapy in all situations with clear indications, even in cases of major bleeding, as long as it is not a life-threatening intracranial or extracranial bleeding [4]. Our findings could contribute to facilitating communication between physicians and patients not only to consider the patient’s preferences but also to convey the physicians’ recommendations to each other. For such points, a recent clinical guideline recommended shared decision-making to explore patients’ values, goals, and preferences [32]. In contrast, all clinical guidelines for patients with decreased kidney function suggest dose adjustments for drugs including anticoagulants [33, 34]. Most of them have not suggested resuming anticoagulants after bleeding events. A recent expert consensus report recommended the resumption of anticoagulation after bleeding events [35]. Considering this lack of coherence between recommendations, our study results might be considered as offering significant evidence and providing a direction for future studies on chronic kidney disease patients with anticoagulants.

This study has several limitations. First, we used different analytic methods to assess the composite outcome events, which could include inherent problems. However, our results demonstrated that the effects of the differences in kidney function on the outcome events were consistent with those from previous studies. Second, this study was performed as a subanalysis of the AFIRE trial. In the original study protocol, the resumption of antithrombotic therapy after a bleeding event was expected to be as early as possible [12]. Furthermore, we did not include the dosages of the drugs used in this analysis. We have assumed that the subsequent decision to continue or discontinue the drugs after bleeding episodes would be similar between the two groups. In each case, these decisions were left to the physicians, which might not be consistent in all patients. However, it is unrealistic to use a unified protocol following bleeding events in future trials. Third, the study population included only Japanese patients with stable coronary artery disease, limiting the generalizability of our findings only to Japanese patients. However, most recent antithrombotic drugs called NOACs have similar renal excretion; therefore, our inferences might be consistent for different NOACs. Fourth, we used CrCl to estimate GFR and the cutoff point at 50 mL/min to succeed for the original AFIRE study protocol. Using other measures to show the kidney function classification might have been more relevant, such as CKD-EPI (Chronic Kidney Disease Epidemiology Collaboration) equations and CKD staging of KDIGO (Kidney Disease Improving Global Outcomes). However, given the imprecision in measures for estimating kidney function, individualization of the drug dosing based on each method is reasonable [33]. Moreover, in our sensitivity analysis, we have also employed the CKD-EPI equation and different cutoff points. Accordingly, we had confirmed the robustness of our findings where different methods of estimates ultimately led to similar results. Finally, our present study results were from prespecified subgroup analyses in addition to being a set of post hoc analyses after a randomized controlled clinical trial; it would be inevitable they are subject to inflated false-positive rates, from multiple testing [36]. Therefore, our results should be interpretated with caution, and future research would be expected; despite this, our findings might not necessarily change significantly.

## Conclusions

In conclusion, our results reaffirmed that kidney function affects bleeding events in patients on antithrombotic therapy, considering recurrent events. We believe that patients on antithrombotic therapy, especially those with decreased kidney function, should receive detailed explanations from their physicians regarding possible bleeding events when continuing antithrombotic therapy.

## Supplementary Information

**Additional file 1: Figure S1.** Estimated risk of bleeding events, CKD-EPI equation at 50 ml/min/1.73m^2^. Time since enrollment (days).

**Additional file 2: Figure S2.** Estimated risk of bleeding events, CKD-EPI equation at 45 ml/min/1.73m^2^. Time since enrollment (days).

**Additional file 3: Figure S3.** Estimated risk of bleeding events, Cockcroft-Gault equation at 45 ml/min. Time since enrollment (days).

**Additional file 4: Figure S4.** Estimated risk of bleeding events, CKD-EPI equation at 60 ml/min/1.73m^2^. Time since enrollment (days).

**Additional file 5: Figure S5.** Estimated risk of bleeding events, Cockcroft-Gault equation at 60 ml/min. Time since enrollment (days).

## Data Availability

Data are available from the corresponding author upon reasonable request.

## Abbreviations

AF: Atrial fibrillation
AFIRE: Atrial Fibrillation and Ischemic Events with Rivaroxaban in Patients with Stable Coronary Artery Disease trial
CG: Cockcroft–Gault
CI: Confidence interval
CKD-EPI: Chronic Kidney Disease Epidemiology Collaboration
CrCl: Creatinine clearance
eGFR: Estimated glomerular filtration rate
NOACs: Non-vitamin K antagonistic oral anticoagulants
