# Supplementary figures and images for: The impact of kidney function in patients on antithrombotic therapy: a post hoc subgroup analysis focusing on recurrent bleeding events from the AFIRE trial
Source: BMC Med. 2022 Feb 25;20:69. doi: 10.1186/s12916-022-02268-6 (PMC8876785; doi:10.1186/s12916-022-02268-6)

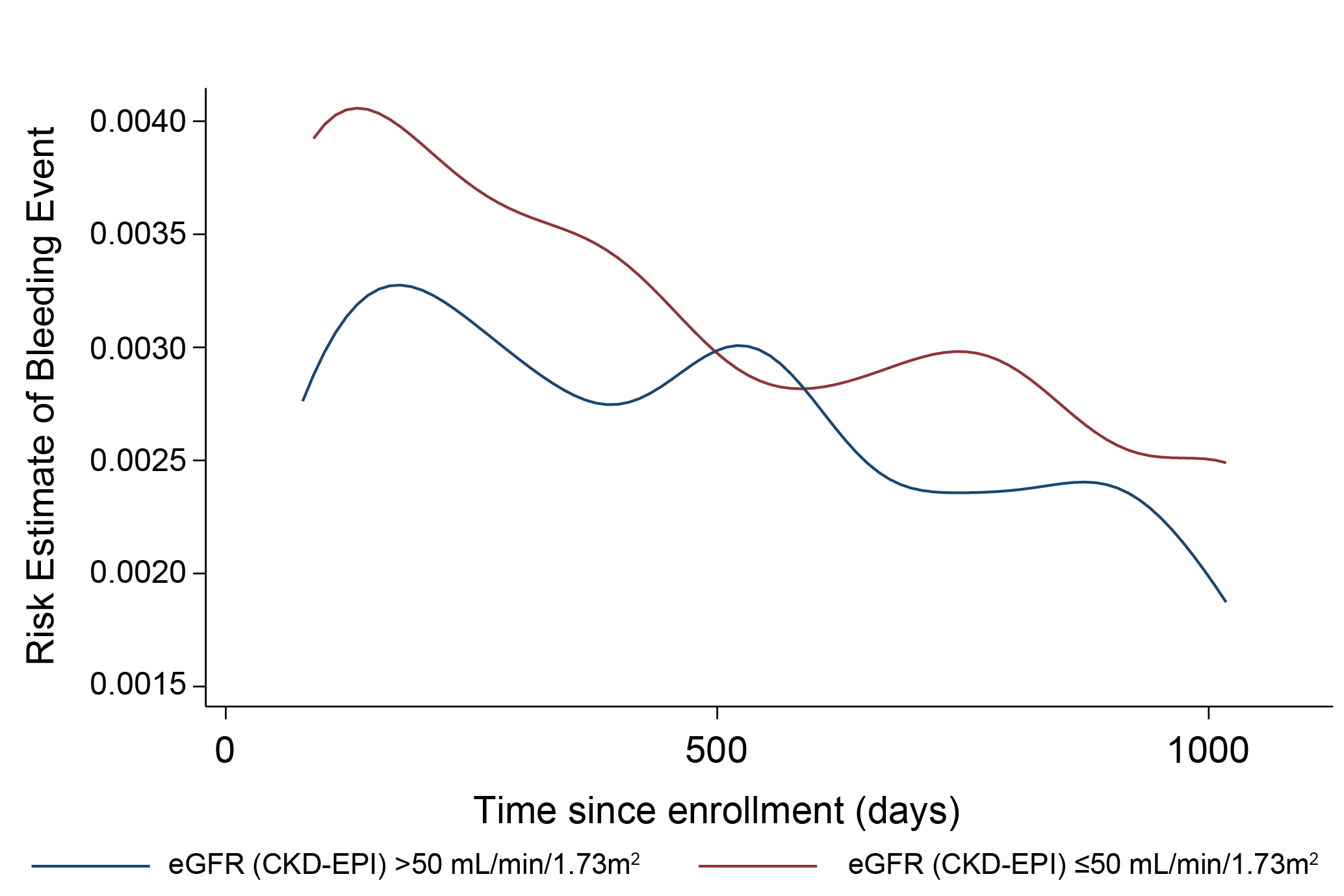

Supplement: Supplementary file 1 — Additional file 1: Figure S1. Estimated risk of bleeding events, CKD-EPI equation at 50 ml/min/1.73m2. Time since enrollment (days). [file 12916_2022_2268_MOESM1_ESM.tif]

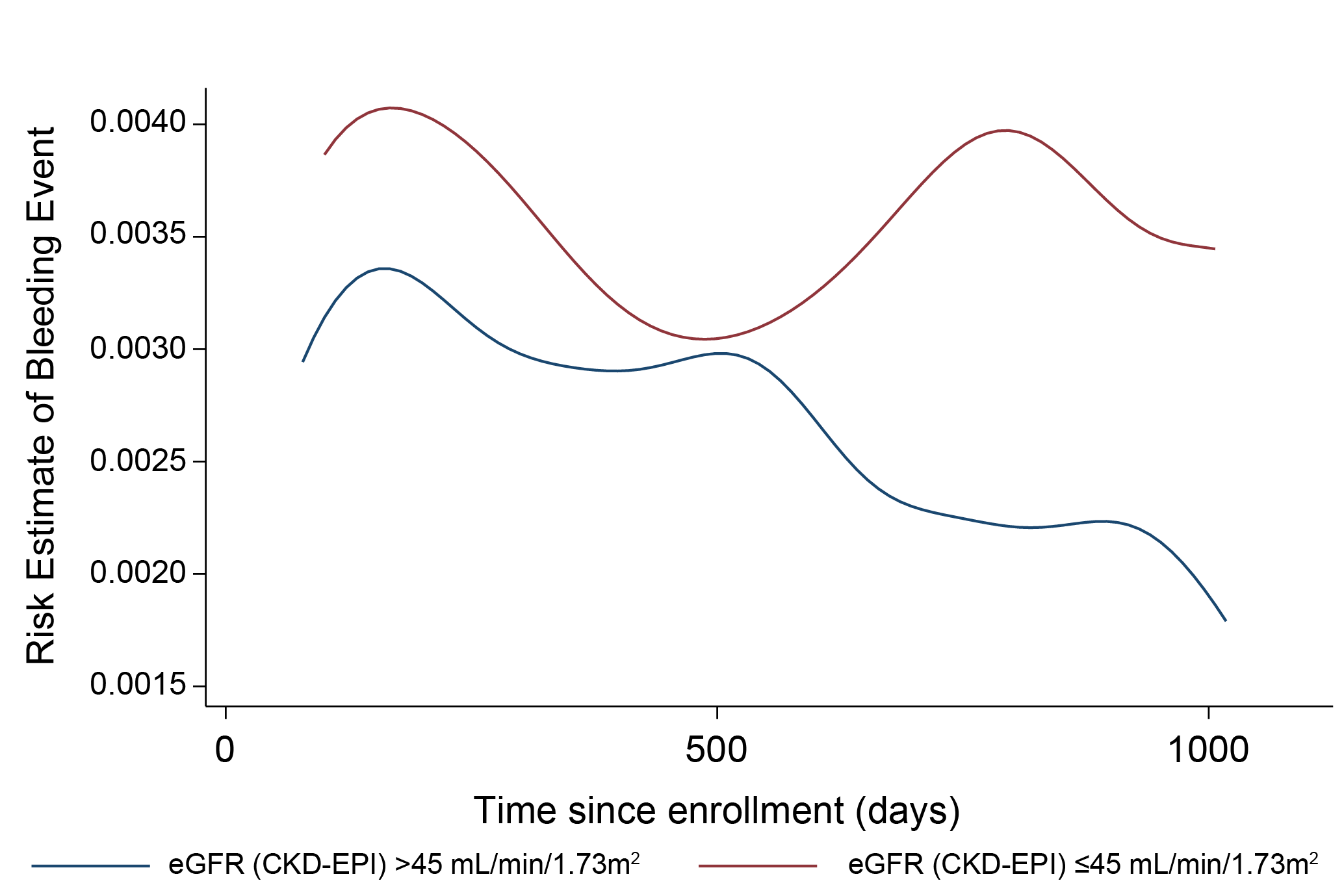

Supplement: Supplementary file 2 — Additional file 2: Figure S2. Estimated risk of bleeding events, CKD-EPI equation at 45 ml/min/1.73m2. Time since enrollment (days). [file 12916_2022_2268_MOESM2_ESM.tif]

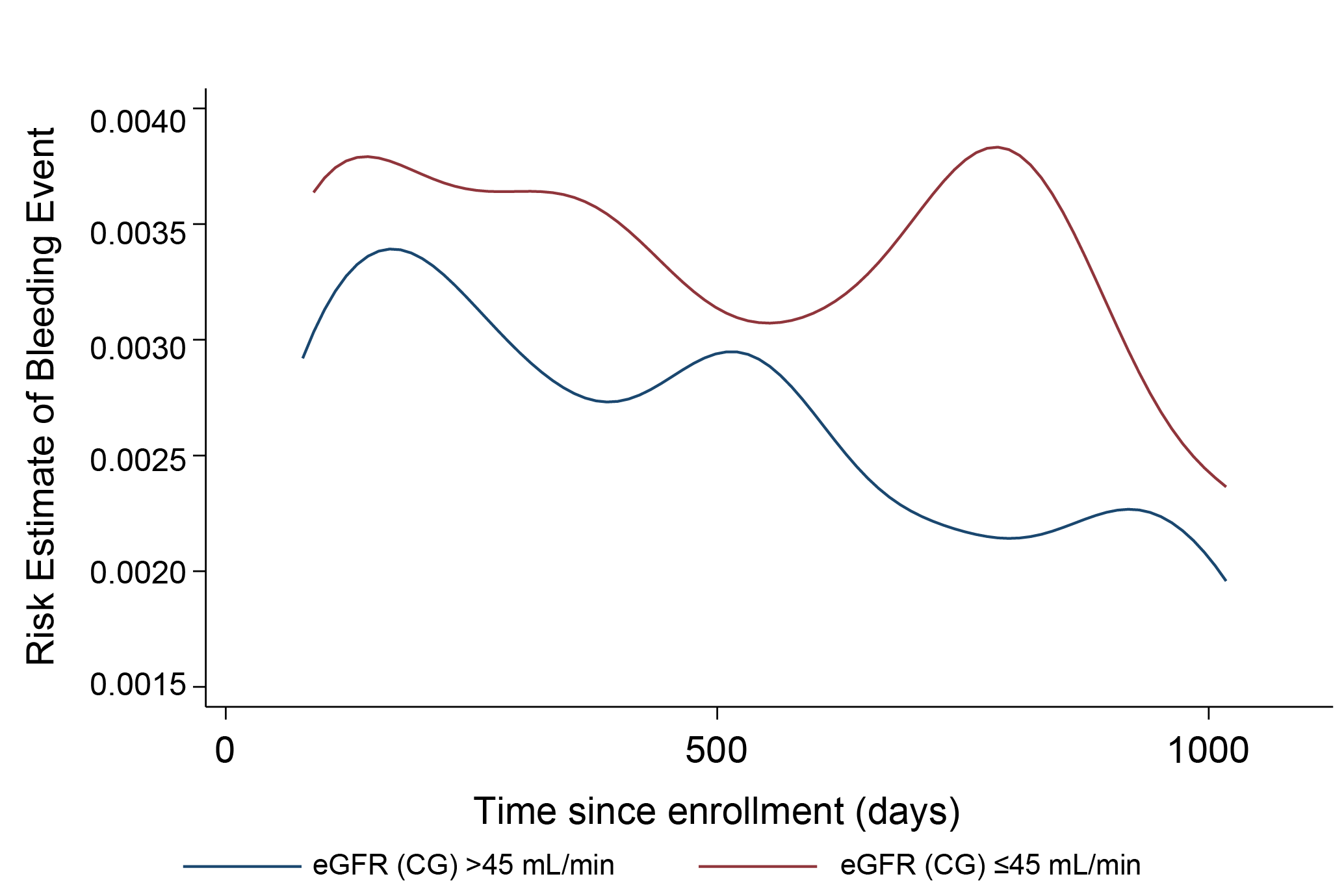

Supplement: Supplementary file 3 — Additional file 3: Figure S3. Estimated risk of bleeding events, Cockcroft-Gault equation at 45 ml/min. Time since enrollment (days). [file 12916_2022_2268_MOESM3_ESM.tif]

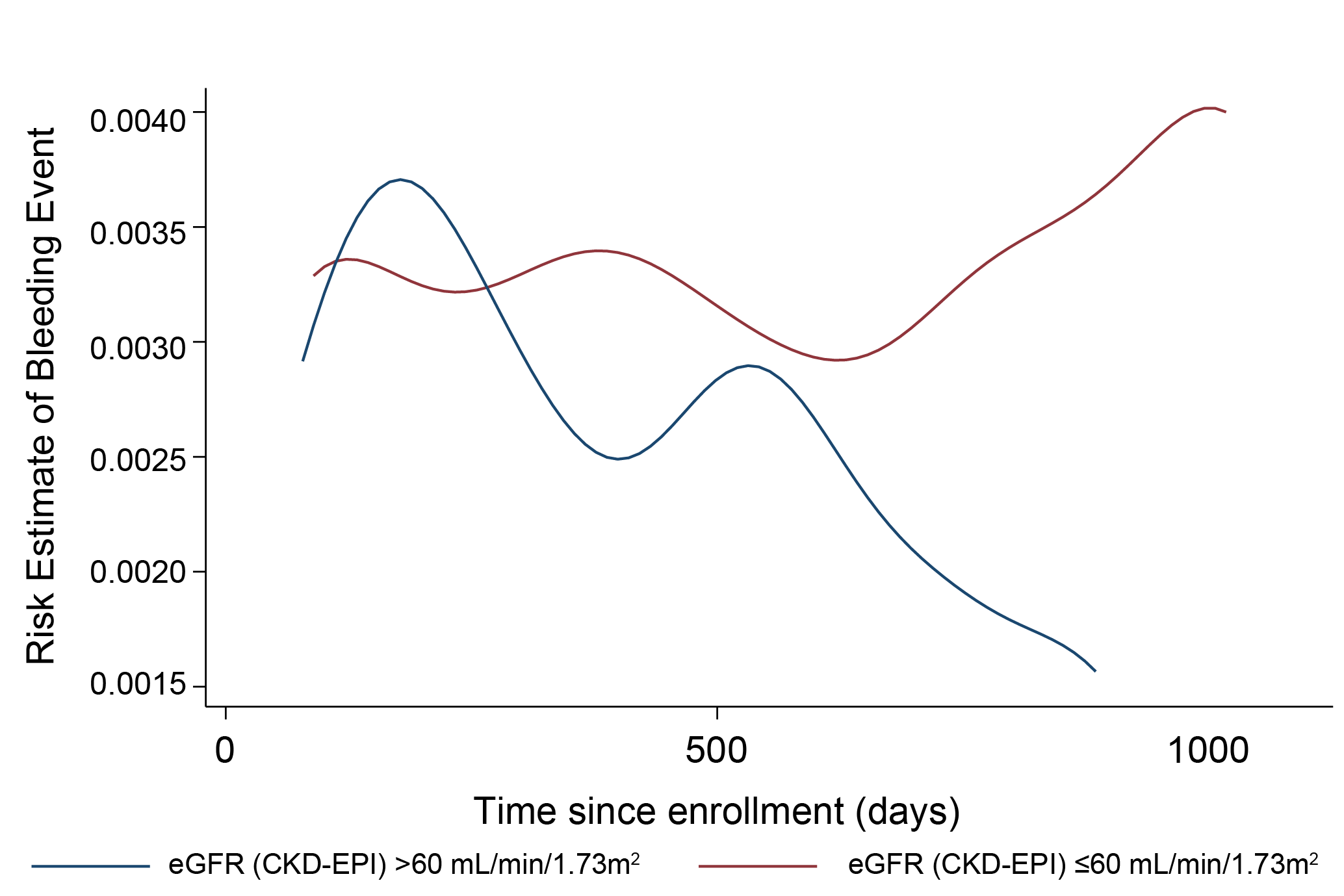

Supplement: Supplementary file 4 — Additional file 4: Figure S4. Estimated risk of bleeding events, CKD-EPI equation at 60 ml/min/1.73m2. Time since enrollment (days). [file 12916_2022_2268_MOESM4_ESM.tif]

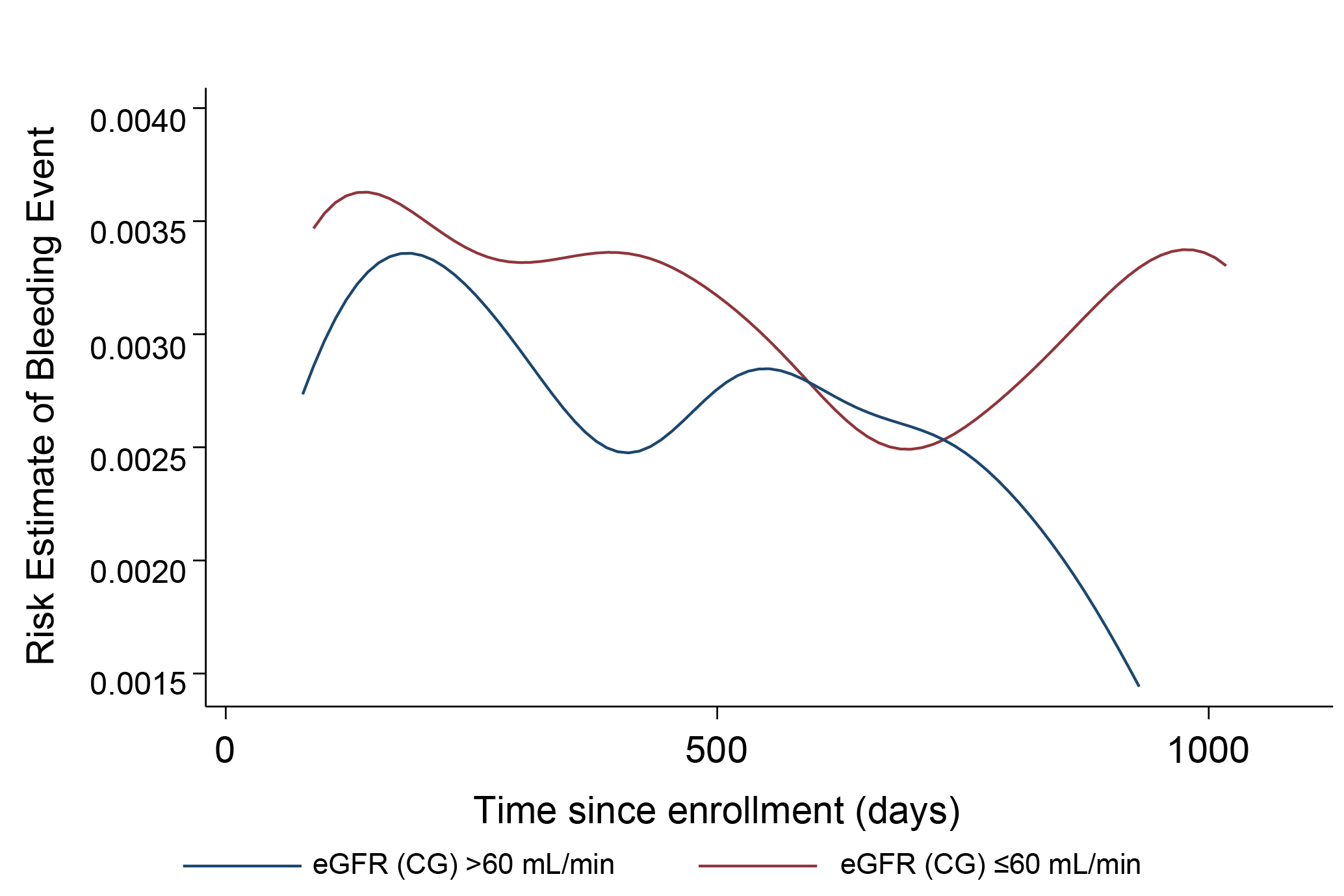

Supplement: Supplementary file 5 — Additional file 5: Figure S5. Estimated risk of bleeding events, Cockcroft-Gault equation at 60 ml/min. Time since enrollment (days). [file 12916_2022_2268_MOESM5_ESM.tif]
